# Supplementary material for: Ultrasensitive antibody-aptamer plasmonic biosensor for malaria biomarker detection in whole blood
Source: Nat Commun. 2020 Dec 1;11:6134. doi: 10.1038/s41467-020-19755-0 (PMC7708447; doi:10.1038/s41467-020-19755-0)
Supplement: Supplementary file 1 — Supplementary Information [file 41467_2020_19755_MOESM1_ESM.pdf]

Supplementary Information

Ultrasensitive antibody-aptamer plasmonic biosensor for malaria biomarker detection in whole blood

Antonio Minopoli[1,2], Bartolomeo Della Ventura[2], Bohdan Lenyk[1,3], Francesco Gentile[4], Julian A. Tanner[5], Andreas Offenhäusser[1], Dirk Mayer[1*], Raffaele Velotta[2*]

[1]Institute of Biological Information Processing (IBI-3), Bioelectronics, Forschungszentrum Jülich, 52425 Jülich, Germany

[2]Department of Physics "E. Pancini", University Federico II, Via Cintia 26, 80126 Naples, Italy

[3]Department of Physics, University of Konstanz, 78457 Konstanz, Germany

[4]Department of Electrical Engineering and Information Technology, University Federico II, 80126 Naples, Italy

[5]School of Biomedical Sciences, University of Hong Kong

Corresponding authors: rvelotta@unina.it, dirk.mayer@fz-juelich.de

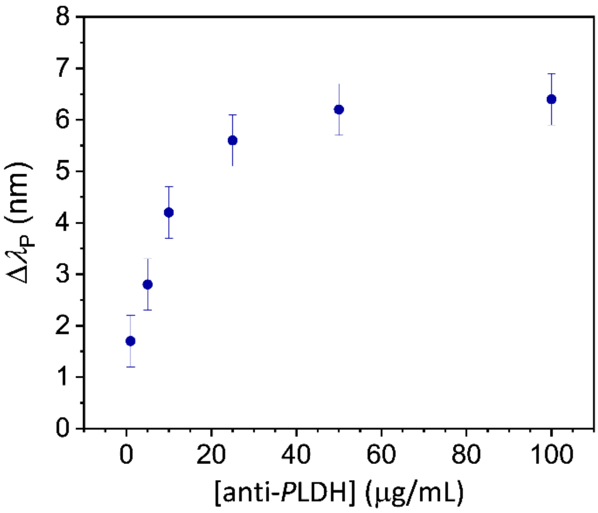

Supplementary Figure 1. Functionalization study. Wavelength shift of the resonance peak as a function of anti-*P*LDH concentration. The functionalization was achieved by flowing 1 mL of irradiated Ab aqueous solution onto the bare substrate. The resonance peak wavelength red-shifted as the anti-*P*LDH concentration increased up to 50 µg/mL that corresponded to a maximum red-shift of 6 nm. For larger amounts of anti-*P*LDH, no change in plasmon resonance wavelength was measured due to the saturation of the gold surface.

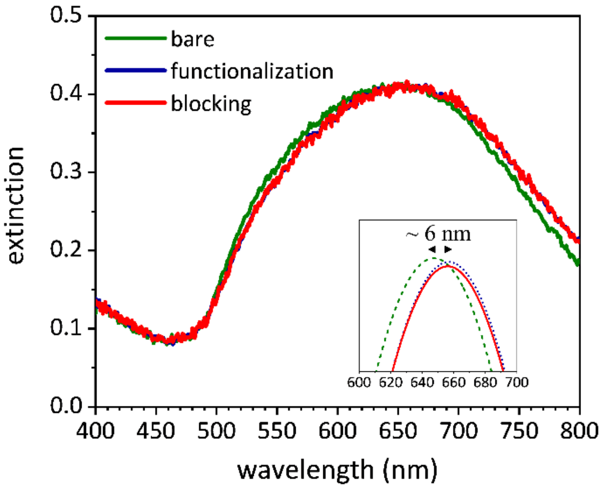

Supplementary Figure 2. Extinction spectra of bare, functionalized and blocked substrate. Bare 2D AuNP array exhibits a broad extinction spectrum peaked at 650 nm (green curve). The red-shift of the plasmon resonance wavelength after the functionalization (~ 6 nm) is due to the coverage of the AuNPs with a dielectric shell of approximately 5 nm thickness. No significant change was observed after the blocking step (red curve) as an evidence of the close-packed functionalization warranted by PIT. The inset shows the Gaussian fit used for evaluating the resonance wavelength for bare (green dashed line), functionalized (blue dotted line) and blocked (red continuous line) substrate.

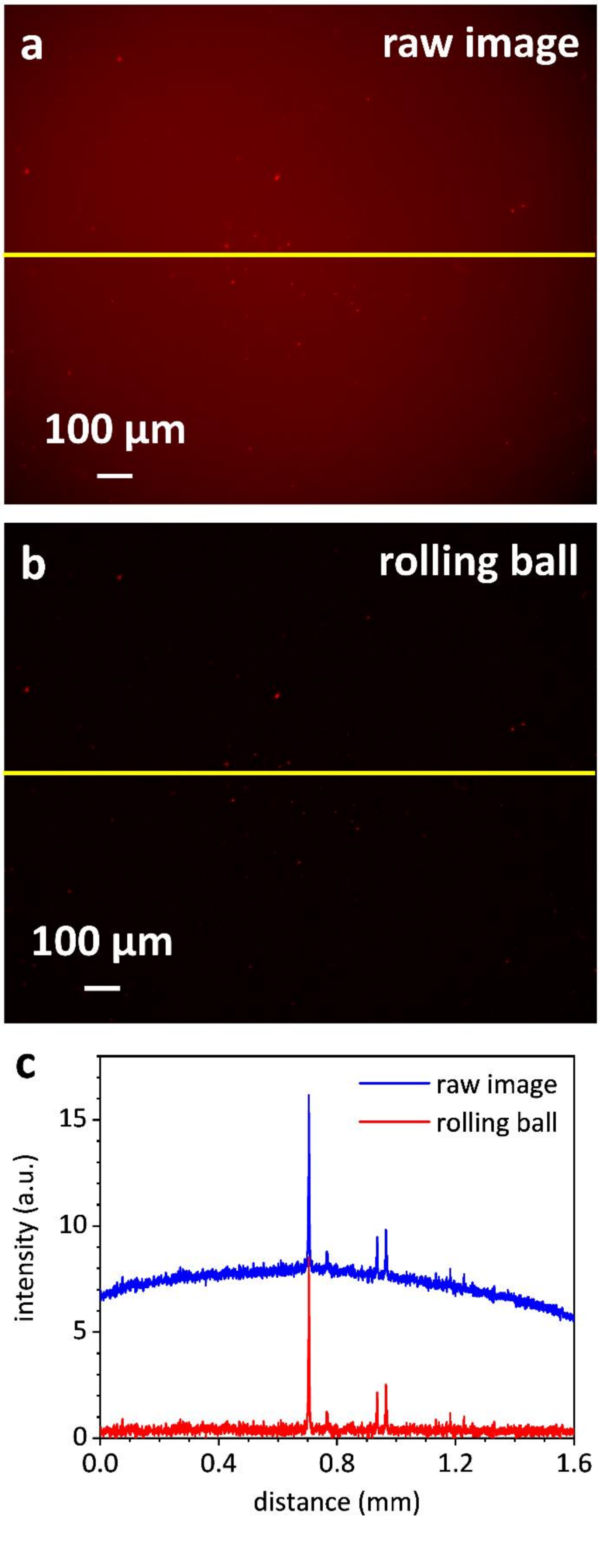

Supplementary Figure 3. Fluorescence image processing. a Example of raw fluorescence image recorded at low *Pf*LDH concentration. The significantly high non-flat background is removed by "rolling ball" algorithm that locally measures the background by averaging over a ball around each pixel and then subtracts such a value from the raw image. The "rolling ball" algorithm reduces the number of false negatives in the case of smooth continuous background as compared to a simple thresholding. **b** Resulting image exhibits a flat background, from which the fluorescence spots are well distinguishable. **c** Intensity profile evaluated along the yellow line highlighted in panels **a** and **b**.

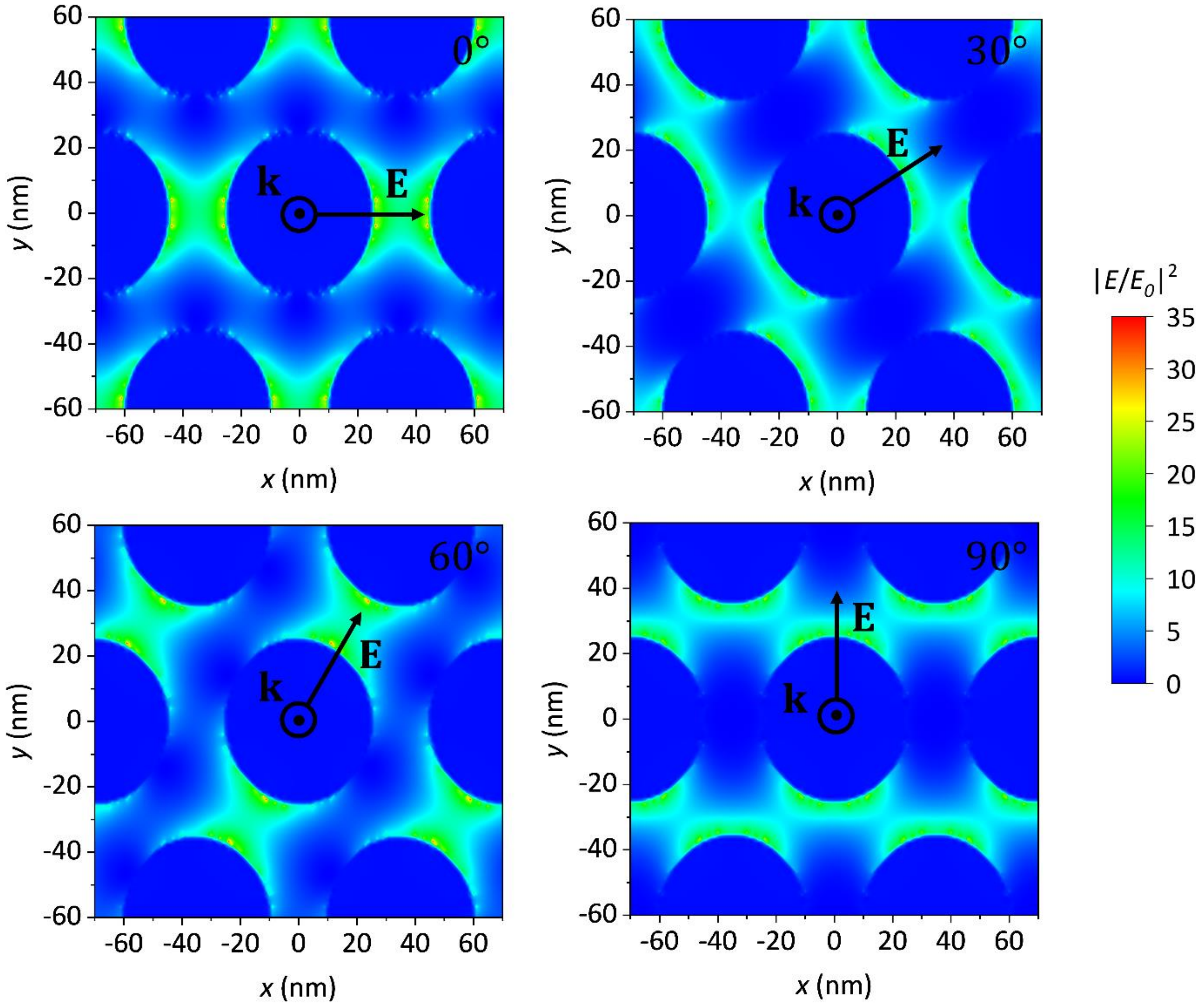

Supplementary Figure 4. FDTD simulations of the E-field intensity. Gain of the E-field intensity provided by a 2D AuNP honeycomb lattice (D = 50 nm, d = 20 nm) as a function of the polarization direction worked out in the x-y plane (z = 0). The highest enhancement is achieved when the polarization direction of the interacting E-field intersects the hexagon vertices.

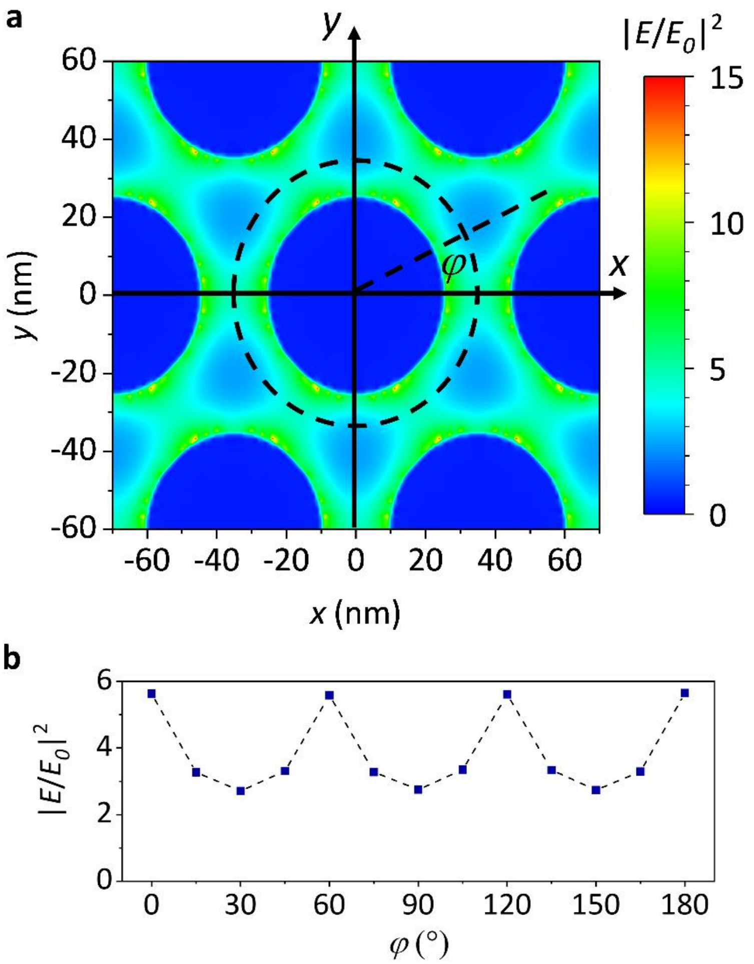

Supplementary Figure 5. E-field intensity of 2D AuNP honeycomb lattice interacting with non-polarized light. a Enhancement of the E-field intensity worked out by averaging the optical response of the lattice (D = 50 nm, d = 20 nm) obtained by varying the polarization angle of the interacting E-field from 0° to 165° with a step of 15°. **b** Modulation of the gain factor as a function of azimuthal angle φ.

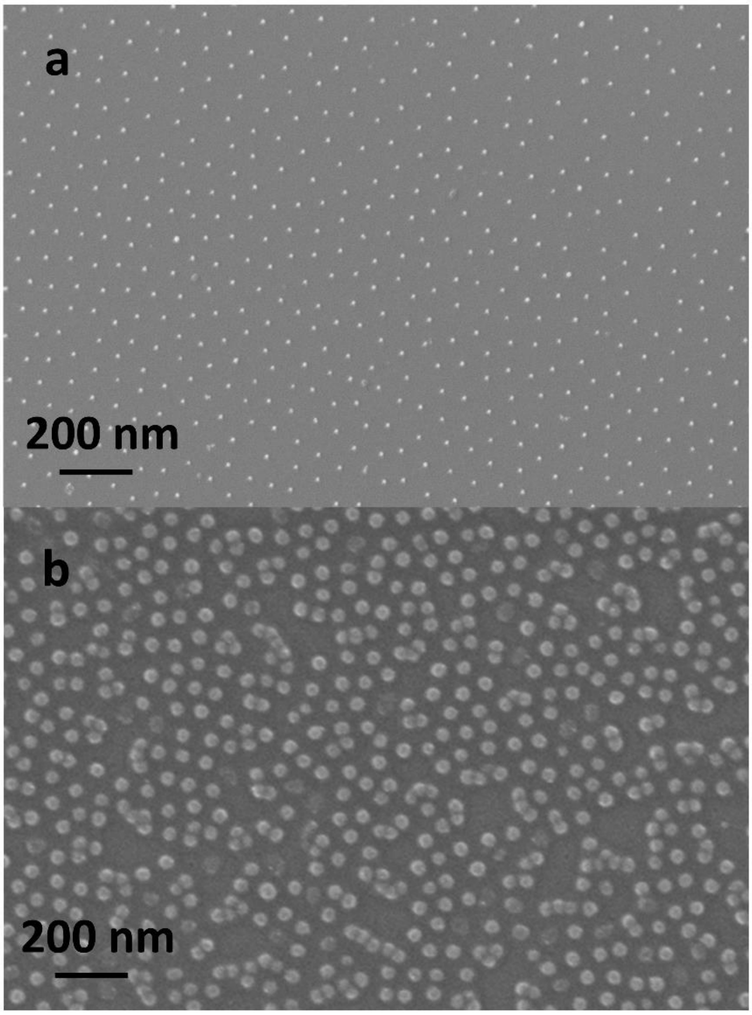

Supplementary Figure 6. SEM images at low magnification. Top view SEM images of the 2D AuNP array **a** before AuNP growth and **b** after 120 min of growth. BCMN warrants high regularity held at macroscopic level; some lattice defects (e.g. clusters and vacancies), randomly distributed on the substrate, arise during the AuNP growth step.

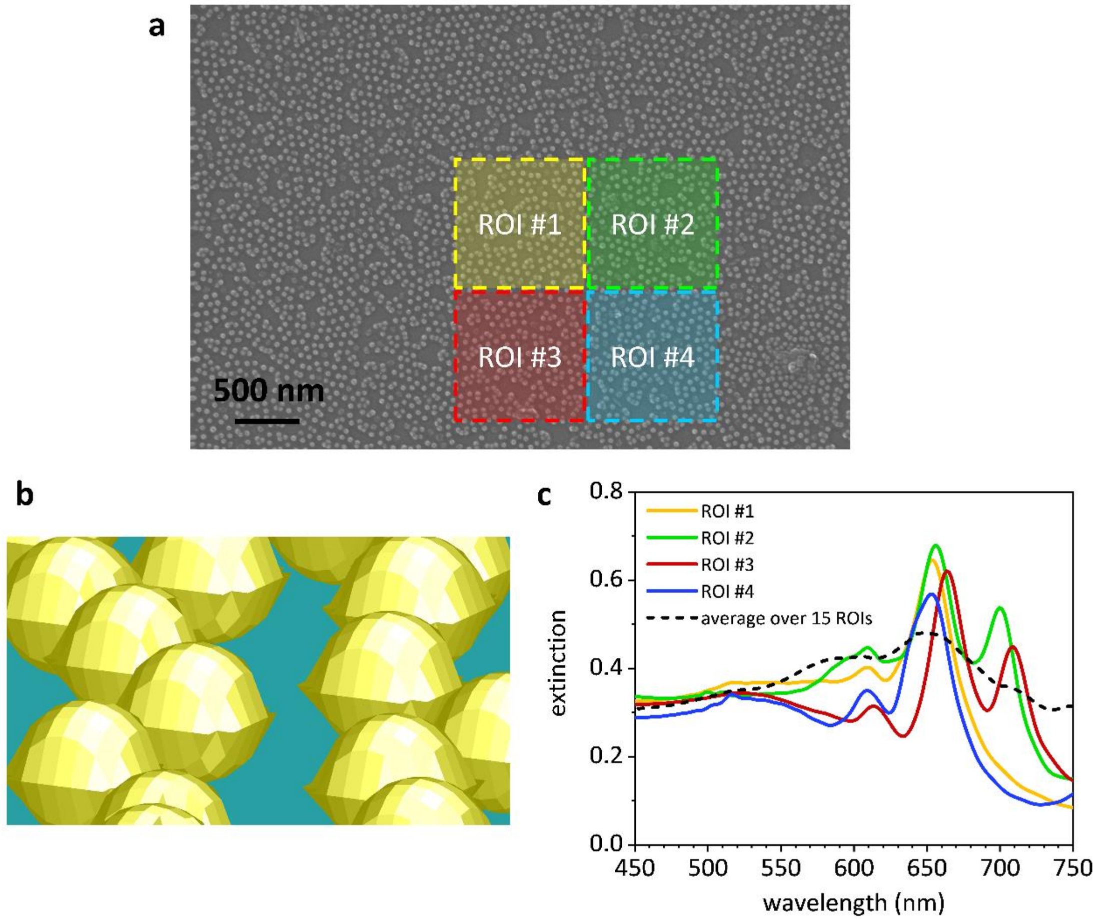

Supplementary Figure 7. Simulated extinction spectra. a Top view SEM images at low magnification. The highlighted squares (1×1 μm^2) identify four out the fifteen regions of interest (ROIs), in which the optical response was simulated. **b** Detail of the substrate rendering provided by Lumerical software. **c** Example of simulated extinction spectra worked out by considering the actual substrate morphology. Line colours are used to identify the corresponding ROIs in panel **a** for which the optical response was investigated.

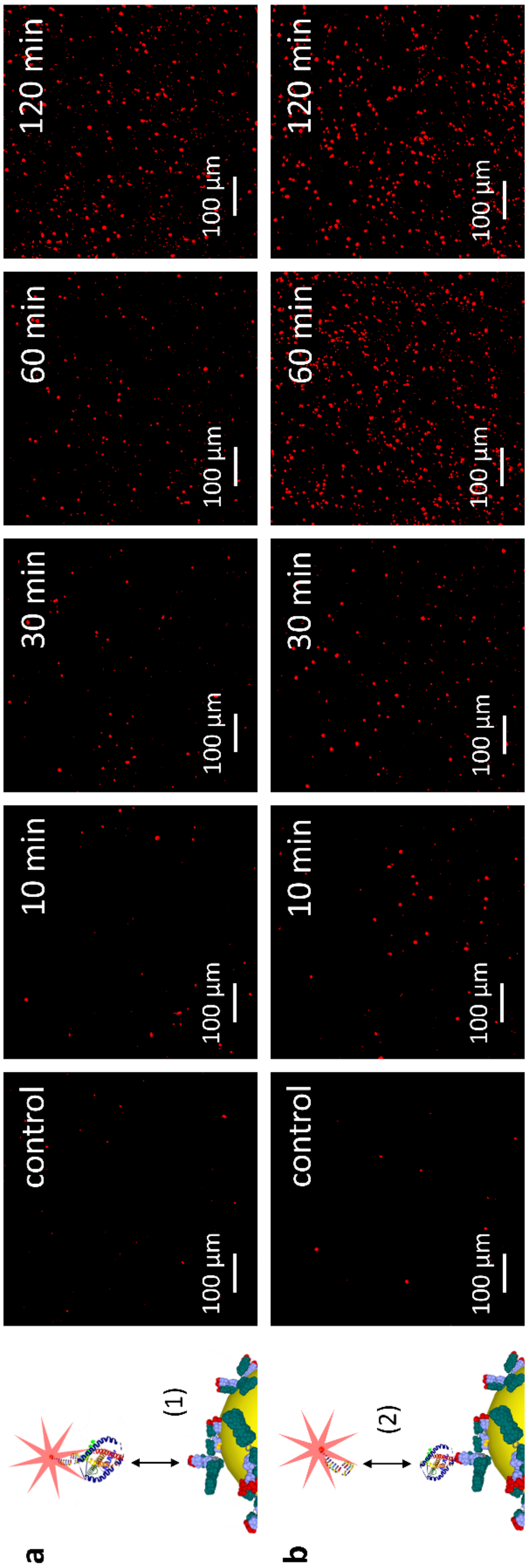

Supplementary Figure 8. Binding kinetic studies. Fluorescence images recorded at different incubation time for both **a** Ab-analyte and **b** aptamer-analyte binding. The number of the fluorescence spots smoothly increases for the process (1) until the reach of the dynamic equilibrium (~80 min), whereas a striking increase of the fluorescence signal is recorded after 30 min for the process (2).

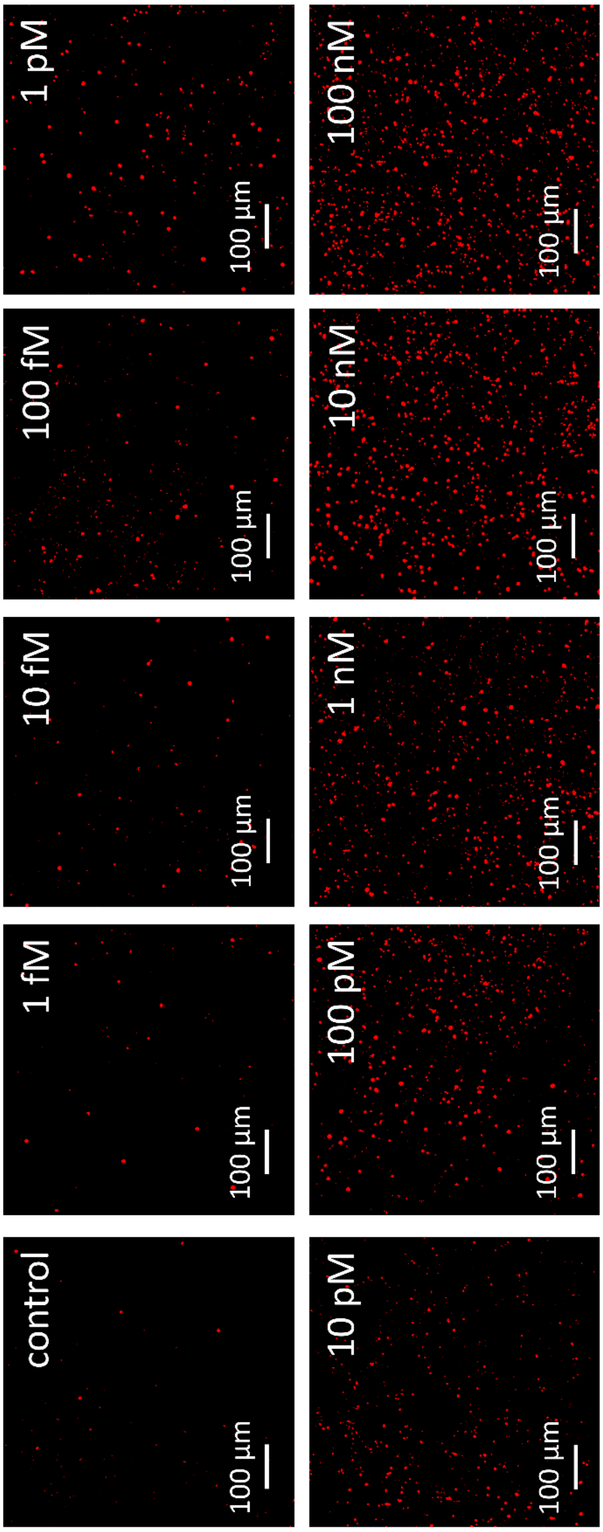

Supplementary Figure 9. Dose-response curve. Example of fluorescence images recorded at different *Pf*LDH concentrations spiked into uninfected whole blood. The number of the fluorescence spots linearly increases going from *Pf*LDH concentration of 10 fM to 10 nM. While no significant change is recorded at 1 fM compared to the control, the number of spots strikingly increases going to higher *Pf*LDH concentrations (≥ 10 fM).

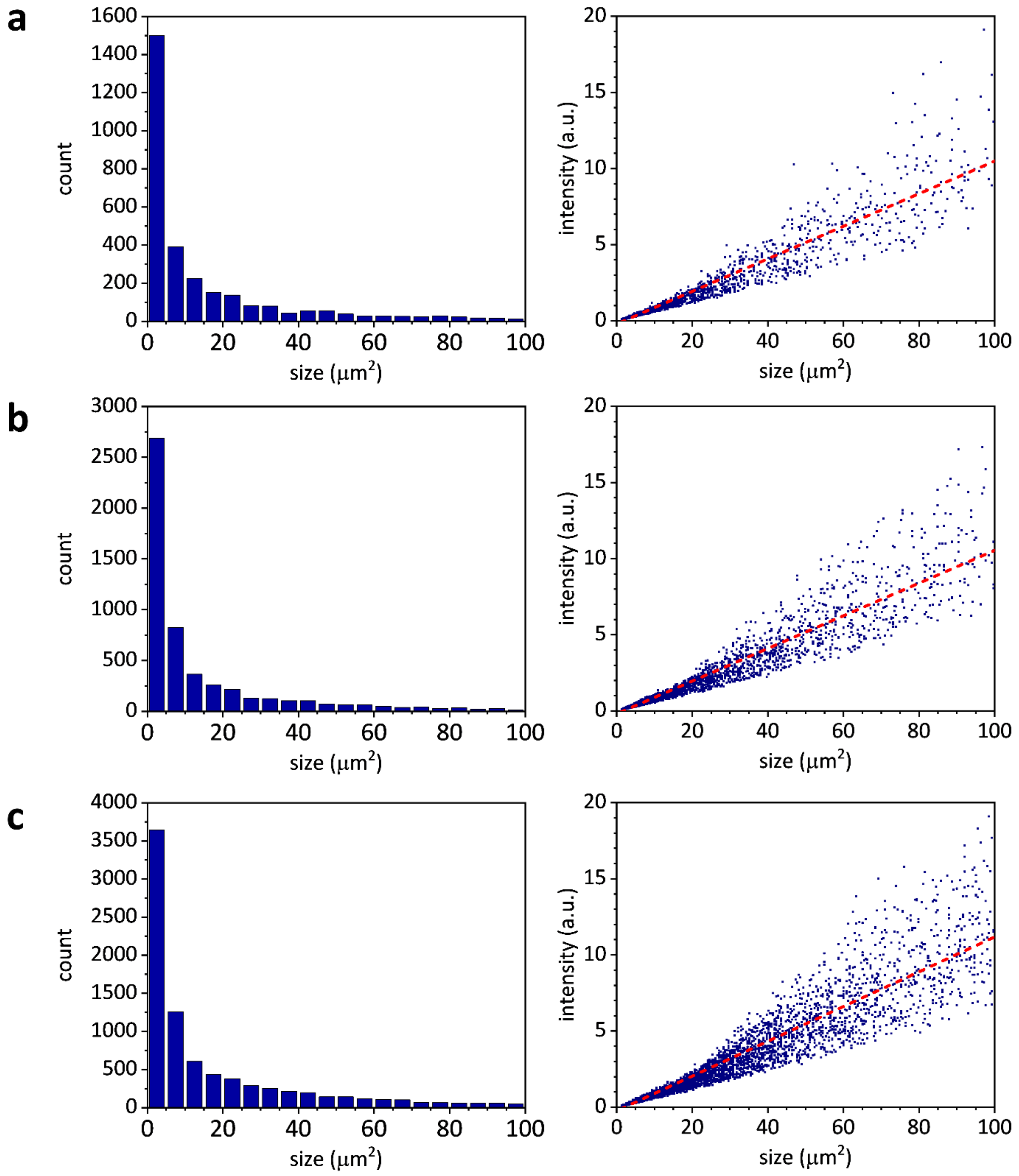

Supplementary Figure 10. Fluorescence spot analysis. Spot size distribution and correlation intensity vs. spot area at **a** 100 fM, **b** 100 pM and **c** 100 nM *Pf*LDH concentration in whole blood show that the mean spot area does not depend on the analyte concentration, whereas the fluorescence intensity of the emitter is strongly related (Pearson's $r = 0.95$) to the number of pixels that collect the signal.

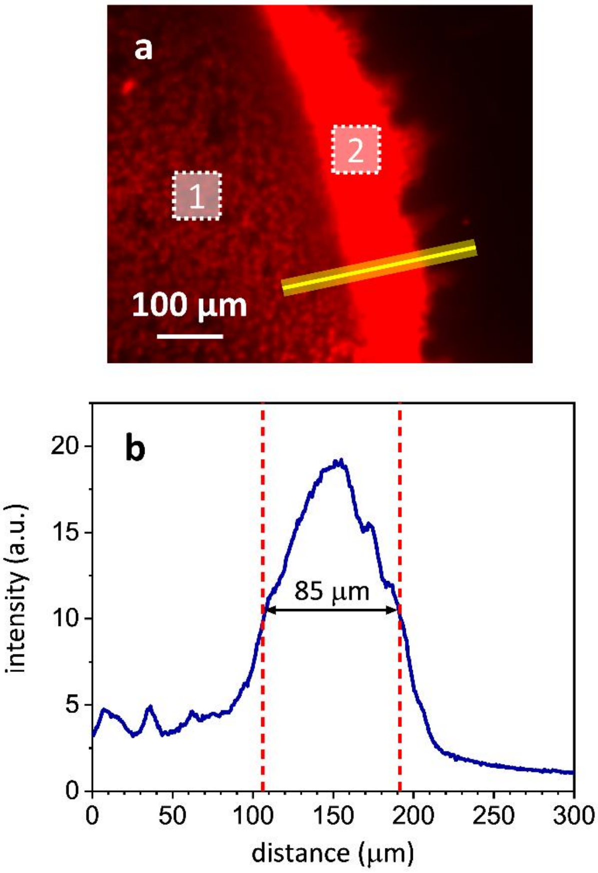

Supplementary Figure 11. Cy5 drop fluorescence intensity. a Example of fluorescence image recorded in close proximity of the drop edge. The dried drop exhibits a circular shape “coffee ring” with a quite uniform inner region (diameter ~12 mm) and an outer annulus whose thickness never exceeds 0.1 mm. The whole intensity of the Cy5 drop was measured by sampling the drop area in one hundred regions of interest (350 × 350 μm^2 in the inner region (white square 1, not in scale) and 70 × 70 μm^2 on the annulus (white square 2)). **b** Ring thickness measured as FWHM of the intensity profile evaluated along the yellow line highlighted in panel **a**.

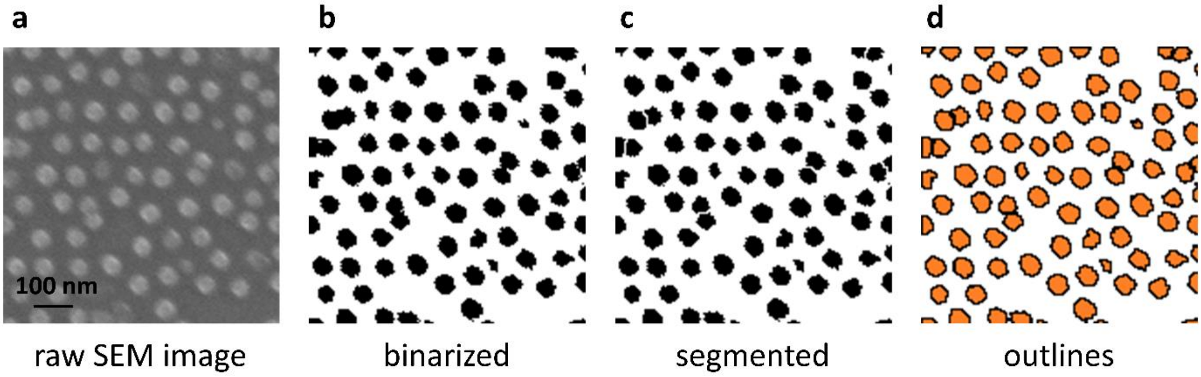

Supplementary Figure 12. SEM image analysis. a Example of raw SEM image at high magnification. Corresponding **b** binarized and **c** segmented image obtained by “Make Binary” and “Watershed” tools, respectively, implemented in ImageJ software. **d** The processing and analysis procedure performed by “Analyze Particle” tool implemented in ImageJ software allows to decompose the objects in outlines (black line) and inner region (orange filling) retrieving morphological information such as object area, perimeter, aspect ratio, circularity and centroid coordinates.

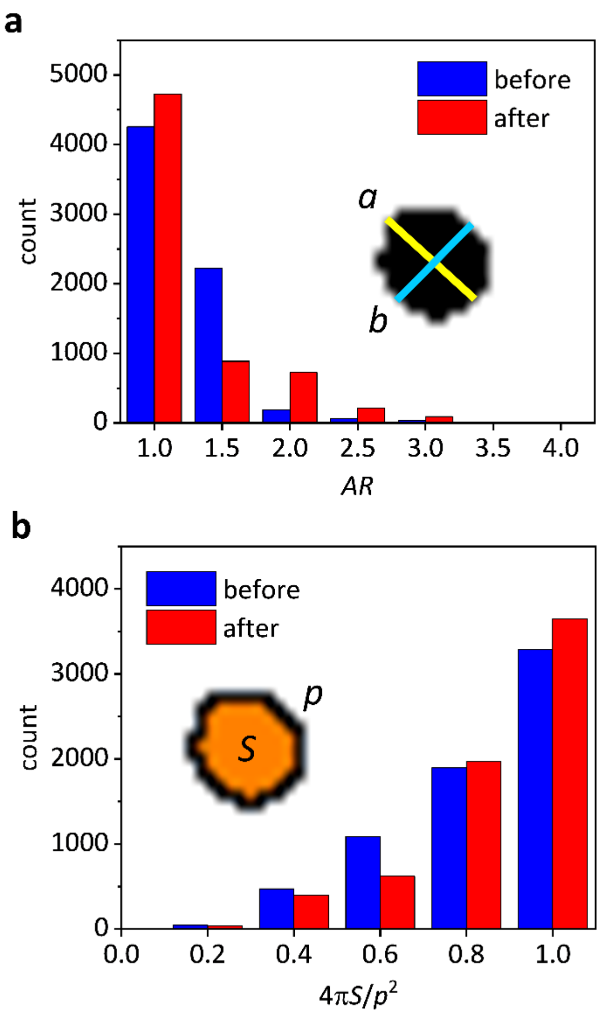

Supplementary Figure 13. Aspect ratio and circularity of immobilized AuNPs. a Histograms of nanoparticle aspect ratio before (blue columns) and after (red columns) incubation with gold growth solution. Object aspect ratio is calculated as $AR = a/b$, where a and b are the major (yellow line) and minor (light blue line) axis, respectively. **b** Histograms of nanoparticle circularity – measured as $4\pi S/p^2$ – before (blue columns) and after (red columns) AuNP growth. The quite long tails on the left side of the circularity distributions are due to the image pixilation that overestimates the perimeter (see the inset).

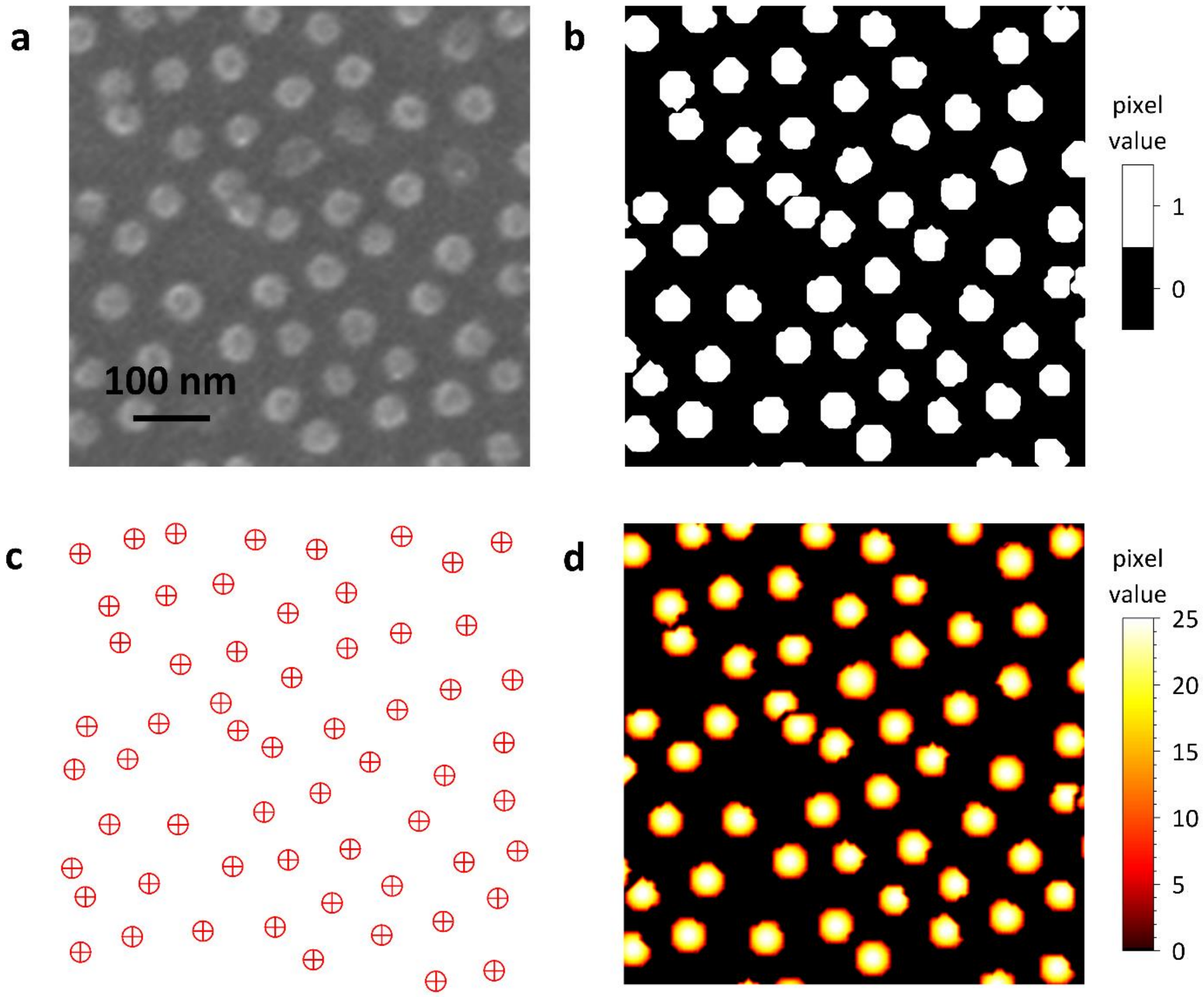

Supplementary Figure 14. Nanoparticle template used for simulations. a Example of raw SEM image at high magnification. **b** Mask template retrieved by thresholding, binarizing and segmenting the SEM image. **c** Centroid mask delivered by running "Analyze Particles" tool implemented in ImageJ software on the binarized and segmented SEM image. **d** Nanoparticle template imported in Lumerical software (see Supplementary Figure 7b for the rendering). Gold nanoparticles were shaped by considering the centroid coordinates shown in panel **c**.
